# High-Resolution Single-Molecule Fluorescence Imaging of Zeolite Aggregates within Real-Life Fluid Catalytic Cracking Particles**

**DOI:** 10.1002/anie.201410236

**Published:** 2014-12-12

**Authors:** Zoran Ristanović, Marleen M Kerssens, Alexey V Kubarev, Frank C Hendriks, Peter Dedecker, Johan Hofkens, Maarten B J Roeffaers, Bert M Weckhuysen

**Affiliations:** Debye Institute for Nanomaterials Science, Faculty of ScienceUtrecht University, Universiteitsweg 99, 3584 CG, Utrecht (The Netherlands); Centre for Surface Chemistry and Catalysis, Faculty of Bioscience EngineeringKU Leuven, Kasteelpark Arenberg 23, 3001 Heverlee (Belgium); Department of Chemistry, Faculty of Sciences, KU LeuvenCelestijnenlaan 200 F, 3001 Leuven (Belgium)

**Keywords:** Brønsted acidity, fluid catalytic cracking, fluorescence, single-molecule microscopy, zeolites

## Abstract

Fluid catalytic cracking (FCC) is a major process in oil refineries to produce gasoline and base chemicals from crude oil fractions. The spatial distribution and acidity of zeolite aggregates embedded within the 50–150 μm-sized FCC spheres heavily influence their catalytic performance. Single-molecule fluorescence-based imaging methods, namely nanometer accuracy by stochastic chemical reactions (NASCA) and super-resolution optical fluctuation imaging (SOFI) were used to study the catalytic activity of sub-micrometer zeolite ZSM-5 domains within real-life FCC catalyst particles. The formation of fluorescent product molecules taking place at Brønsted acid sites was monitored with single turnover sensitivity and high spatiotemporal resolution, providing detailed insight in dispersion and catalytic activity of zeolite ZSM-5 aggregates. The results point towards substantial differences in turnover frequencies between the zeolite aggregates, revealing significant intraparticle heterogeneities in Brønsted reactivity.

Fluid catalytic cracking (FCC) is a major industrial process to convert crude oil into gasoline and valuable hydrocarbons, such as propylene.[1–3] In this catalytic process 50–150 μm-sized spherical particles are used, which contain an acidic zeolite embedded in a matrix of clay, silica, and alumina. The zeolite components with acidic properties, being either zeolite Y or ZSM-5, play a crucial role in the overall catalytic cracking properties.[4–6] The acidity of zeolite domains changes during catalyst activation and aging, but a detailed characterization of the acidity distribution within a single FCC catalyst particle has proven to be extremely difficult due to their intrinsic chemical and structural complexity.

More recently, FCC catalyst particles have been the subject of detailed studies at the single-particle level. Confocal fluorescence microscopy (CFM) in combination with acid-catalyzed staining reactions was used to visualize the dispersion of zeolites Y and ZSM-5 domains within FCC particles.[7–9] By integrating a fluorescence microscope within a transmission electron microscope Karreman et al. have been able to correlate the changes in acidity as probed with fluorescence microscopy, with structural changes and damage.[10] Using the CFM approach, complemented by the results of X-ray microscopy techniques,[11] it is now possible to evaluate the aging process of the catalyst because of the metal deposition (poisoning) and steaming (dealumination). Unfortunately, CFM cannot resolve sub-micrometer zeolite domains and does not provide the quantitative information about catalytic activity of individual zeolite aggregates.

Single-molecule fluorescence microscopy has emerged as a very sensitive and informative technique in life sciences, and more recent efforts have directed to extend this powerful approach to the field of materials science, including but not limited to homogeneous and heterogeneous catalysis.[12–17] The single-molecule sensitivity of fluorescence detection is used to enhance the spatial resolution down to the nanometer scale, making it an ideal candidate for assessing the reactivity of catalytic solids. The technique has been used for studies on well-defined heterogeneous catalysts; for example, single-molecule kinetics of nanoparticle catalysts,[18–20] and high-resolution imaging of catalytic activity in porous heterogeneous catalysts.[21–23]

Here we report the first application of single-molecule fluorescence microscopy and the required analysis methods to quantitatively study Brønsted-catalyzed reactivity of hierarchically structured, multi-component and industrially applied FCC catalyst particles, containing zeolite ZSM-5 as the active cracking phase. The presented approach can be widely applied to other complex catalyst materials like granulated particles that suffer from an elevated background luminescence. We also anticipate that this approach will broaden the scope of fluorogenic reactions that can be used for quantitative reactivity mapping since it possesses less stringent demands on the fluorophore properties. Since individual catalytic events no longer need to be isolated, measurements with higher catalytic turnover densities can be used for quantitative analysis leading to more information in the same experiment time.

To selectively study the zeolite domains within the FCC catalyst particles we have used the oligomerization of furfuryl alcohol as a probe reaction. This reaction is catalyzed by Brønsted acid sites present within zeolites or metal–organic frameworks and can be used for catalytic reactivity mapping with single turnover sensitivity.[22,24,25] A schematic of the method is shown in Figure 1 a. The 532 nm laser light can efficiently excite fluorescent oligomers that are catalytically formed from non-fluorescent furfuryl alcohol molecules (Figure 1 b; for the experimental details and the mechanism of the probe reaction see sections S1–3 in the Supporting Information).

**Figure 1:**
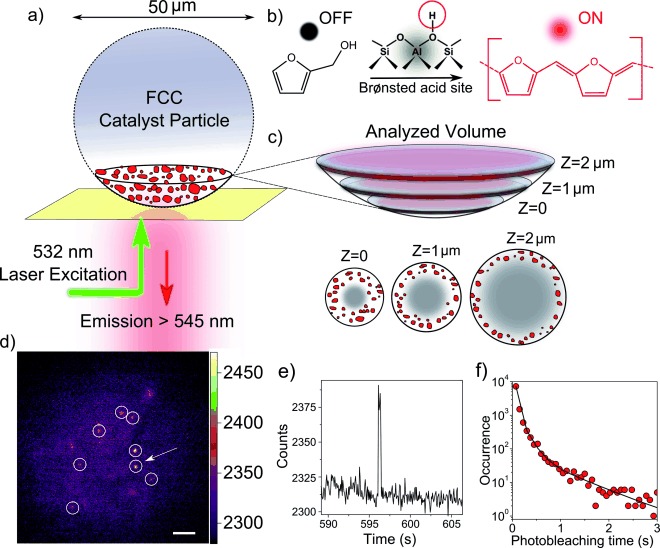
Schematic of the single-molecule fluorescence approach. a) A single FCC catalyst particle, containing zeolite ZSM-5 domains depicted in red, studied with a wide-field fluorescence microscope setup. b) Formation of the fluorescent products (red) upon oligomerization of non-fluorescent furfuryl alcohol (black) on a Brønsted acid site. c) The geometry of the analyzed focal slices and denoted focal depths. The inner regions of the FCC particles (depicted in gray) were not included in the later analysis due to the attenuation of fluorescent light and mass transfer limitations. d) A wide-field fluorescence micrograph of an FCC particle recorded during the oligomerization of furfuryl alcohol (exposure time of 75 ms; scale bar=2 μm). White circles indicate localized fluorescence bursts originating from fluorescent products. e) A photo-trajectory of a representative single catalytic turnover, indicated with a white arrow in Figure 1 d. f) Distribution of measured survival times of about 10 000 fluorescent product molecules before photobleaching, fitted with the multi-exponential function with the exponential time constants of 0.035, 0.15, and 0.74 s.

As the fluorescent products are formed exclusively on Brønsted acid sites, their fluorescence can be used for the 3D localization of zeolite ZSM-5 domains embedded within the matrix material of a single FCC particle (Figure 1 c). The individual fluorescent reaction products are detected with an EM-CCD camera (Figure 1 d). A typical fluorescence intensity trajectory of an individual hotspot is shown in Figure 1 e. It was found that the characteristic survival time of fluorescent products before photobleaching is typically smaller than 0.3 s (Figure 1 f). Therefore, we may conclude that fluctuations in the fluorescence intensity happening at specific locations at the second time scale are mostly caused by the formation of new fluorescent product molecules on acid sites of individual zeolite domains.

Figure 2 a illustrates four isolated fluorescent product molecules localized by fitting their point spread functions (PSF) with a 2D Gaussian (see section S4 in the Supporting Information). This method of localizing stochastic catalytic turnovers in heterogeneous catalysis is commonly known as NASCA microscopy, that is, nanometer accuracy by stochastic chemical reactions microscopy.[22] Recording a fluorescence movie (see Movie S1 in the Supporting Information) allows reconstructing of a high-resolution NASCA image based on the precise localization of individual reaction events, as illustrated in Figure 2 b. However, NASCA measurements typically require well-controlled reaction conditions with a good signal-to-noise ratio and therefore are challenging for industrial catalysts with high structural complexity and intrinsic background fluorescence. Ideally, a complementary method that can operate under less stringent conditions and in a wider range of concentrations is necessary to support the results of the NASCA analysis.

**Figure 2:**
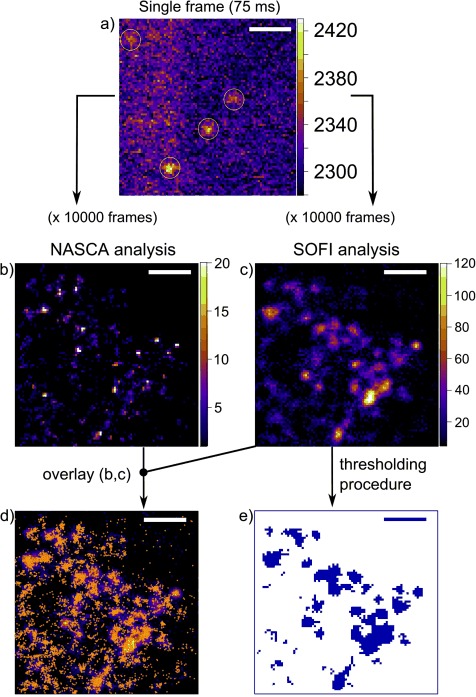
a) A zoom-in 75 ms frame; white circles indicate localized single-molecule fluorescence bursts. b) An accumulated NASCA high-resolution map of individual catalytic turnovers. The color scale represents the number of detected turnovers per pixel (48×48 nm^2^). c) A corresponding SOFI image. The color scale denotes calculated SOFI intensities. d) An overlay of the SOFI image from (c) and localized emitters positions obtained from NASCA analysis presented in (b), yellow circles represent detected catalytic events. e) Reconstructed binary image of the fluorescent regions from (c). The scale bars are 1 μm.

In order to complement the results of the NASCA method we opted to use the super-resolution optical fluctuation imaging (SOFI) analysis—a method that is developed recently for imaging of cellular structures in experiments with low signal-to-noise ratio.[26–29] This method relies on statistical analysis of temporal fluctuations in consecutive fluorescence images to provide essentially background-free, contrast-enhanced images with improved resolution in all three dimensions.[26] In our experiments, independent stochastic fluctuations of fluorescent emitters appear as a result of the constant formation, diffusion and photobleaching of fluorophores taking place at zeolite domains. The recorded signal in the SOFI images is not trivially related to the recorded fluorescence intensity.[26,28] However, it is proportional to the local concentration of fluorophores, provided that they exhibit similar emission properties and fast fluctuations of the fluorescence signal—conditions that are met in our experiment. As an example, Figure 2 c shows an accumulated SOFI image reconstructed based on an identical movie as the NASCA image in Figure 2 b. An overlay of the images based on the two methods in Figure 2 d indicates that the brightest regions in the SOFI image are indeed the ones where most of the fluorescent events and thus catalytic turnovers are recorded.

Using SOFI images as a reference for the stochastic fluctuations of fluorescence signal, we applied a binary thresholding procedure in order to statistically analyze the size of the catalytically most active zeolite domains (Figure 2 e). The procedure separates the fluorescence domains with high intensity in SOFI images from the ones with low signal by setting a threshold value based on a systematic analysis of domain brightness and size (see section S5). The reactivity of an individual FCC catalyst particle is monitored for three different focal depths, close to the surface (*Z*=0±0.3 μm), for *Z*=1 μm, and *Z*=2 μm below the surface (Figure 3). Reconstructed SOFI images are presented in Figure 3. The analysis of the SOFI signal points towards notable attenuation of both the excitation and emission by matrix additives of the FCC particle (section S6). Therefore, in our further analysis only the outer regions of similar brightness are investigated for reliable comparison and subsequent thresholding analysis. After the thresholding procedure, the binary images of highly active fluorescent domains (Figure 3, thresholding) are segmented further to account for pixel artifacts and attenuation of the fluorescence in the inner parts (Figure 3, segmentation). Figure 3 shows the obtained histograms of the size distribution of zeolite domains. Most of the zeolite domains are well-dispersed in the analyzed 3D volume and within the 2D projection size of 0.2 μm^2^, which corresponds to spherical particles of about 500 nm in diameter. A similar distribution of zeolite particle sizes was recently reported in a CFM study, supporting the correctness of our approach.[9] However, the analysis of smaller zeolite domains is hardly possible with CFM because of its intrinsic limitations in resolution and sensitivity (section S7). This observation highlights the real strength of the fluorescence-based single molecule approach reported in this work. The NASCA method can typically localize single catalytic turnovers with 20 nm resolution, while the more broadly applicable SOFI method routinely achieves a spatial resolution of 120 nm.

**Figure 3:**
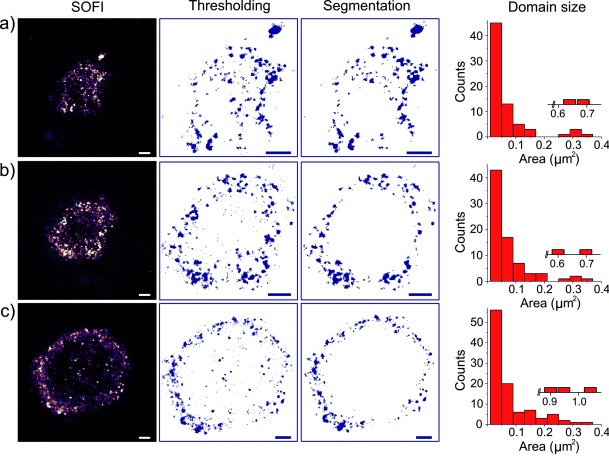
Cluster analysis of zeolite ZSM-5 domains size within a single FCC catalyst particle: a) *Z*=0 (±0.3) μm, b) *Z*=1 μm, c) *Z*=2 μm. Left: SOFI images of the FCC catalyst particle at three different focal depths; a and c) reconstructed based on 10 000 frames movies, and b) reconstructed based on 5000 frames movie. Middle: Corresponding binary images reconstructed based on the threshold analysis and further segmentation. Scale bars are 2 μm. Right: Corresponding histograms of the zeolite domains size distribution. Insets denote single clusters larger than 0.4 μm^2^.

The obtained NASCA and SOFI maps of reactivity indicate that zeolite ZSM-5 domains embedded within a single FCC particle differ in their overall fluorescence activity, which suggests potential differences in their catalytic reactivity. The illustration of this observation is presented in Figure 4 a. The fluorescence intensity trajectories in these regions confirm dependence of SOFI intensity from the catalytic activity of individual domains, such as the regions of high (Figure 4 b), medium (Figure 4 c), and low catalytic activity (Figure 4 d). To support the quantitative aspect of the SOFI analysis we have analyzed 65 zeolite domains localized within a 6×6 μm^2^ region of interest from the SOFI image in Figure 3 a and attempted to correlate their averaged SOFI intensities with corresponding catalytic turnover frequencies. Turnover frequencies of zeolite domains were calculated based on two separate quantification methods. The first method is based on the previously described NASCA approach and Gaussian localization of fluorescent events in 144×144 nm^2^ regions of interest. In the second method, we quantified the number of catalytic turnovers based on fluorescence intensity trajectories of individual zeolite domains (Figure 4 b–d and section S8). Turnover rates of individual zeolite domains are then determined by normalizing the number of detected turnovers by the integration time (750 s) and the size of the regions of interest (144×144 nm^2^).

**Figure 4:**
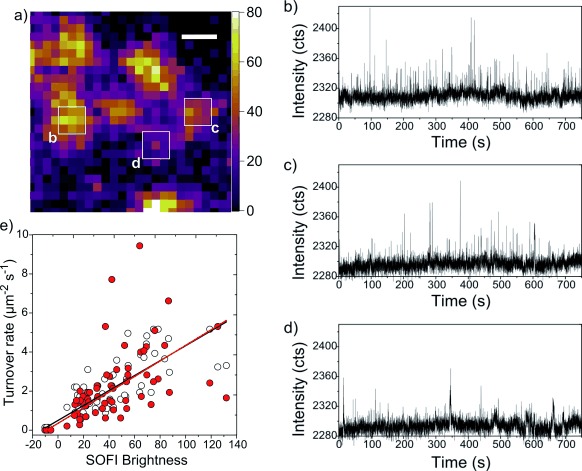
a) SOFI image of a selected 1 μm^2^ region of interest indicating fluorescent zeolite domains. The scale bar is 200 nm. b–d) Fluorescence intensity trajectories for domains labelled in Figure 4 a. e) Catalytic turnover rate as a function of average brightness in the SOFI image, calculated for 65 individual zeolite domains. White circles: values calculated based on fluorescence intensity trajectories; black line is the best linear fit (*R*^2^=0.88, *k*=0.038±0.002). Red circles: values calculated based on Gaussian fitting procedure; red line is the best linear fit (*R*^2^=0.69, k=0.040±0.004).

The comparison of both methods is presented in Figure 4 e. The average brightness of the domains in a SOFI image seems to be in a good approximation proportional to the corresponding numbers of detected catalytic turnovers. The deviation from the linear trend is a consequence of inherent properties of the applied methods. For instance, brighter emitters will have a higher contribution to the intensity of a SOFI image.[26] Figure 4 e suggests that the zeolite domains may differ significantly in their SOFI brightness, thus catalytic reactivity. The average turnover frequency of highly active zeolite domains is calculated to be around five events per second per square micrometer at the studied experimental conditions. This is approximately an order of magnitude difference in activity when compared to the less reactive zeolite ZSM-5 domains. Most probably, the origin of this difference in reactivity is related to differences in framework aluminium content of zeolite domains or local accessibility differences.

In conclusion, single-molecule fluorescence methods proved to be a very sensitive tool to localize with high spatial resolution and single turnover sensitivity the activity of acidic zeolite domains within a hierarchically structured, industrially used FCC catalyst particle. In addition, the explored SOFI analysis emerged as a practical method that can bridge the inherent deficiencies of confocal and single-molecule localization methods, especially in experiments with pronounced intraparticle differences in reactivity and low signal-to-noise ratio. Using the developed analysis approach one could imagine a wide range of in-depth characterization studies of various complex multi-component catalytic materials.
