# Supplementary material for: High-Resolution Single-Molecule Fluorescence Imaging of Zeolite Aggregates within Real-Life Fluid Catalytic Cracking Particles
Source: Angew Chem Int Ed Engl. 2014 Dec 12;54(6):1836–40. doi: 10.1002/anie.201410236 (PMC4506548; doi:10.1002/anie.201410236)
Supplement: Supplementary file 1 [file anie0054-1836-sd1.pdf]

Angewandte Chemie
Eine Zeitschrift der Gesellschaft Deutscher Chemiker

Supporting Information

© Wiley-VCH 2015

69451 Weinheim, Germany

High-Resolution Single-Molecule Fluorescence Imaging of Zeolite Aggregates within Real-Life Fluid Catalytic Cracking Particles**

Zoran Ristanović, Marleen M. Kerssens, Alexey V. Kubarev, Frank C. Hendriks, Peter Dedecker, Johan Hofkens, Maarten B. J. Roeffaers, and Bert M. Weckhuysen**

anie_201410236_sm_miscellaneous_information.pdf
anie_201410236_sm_Movie_S1.avi

Supplementary movie 1

A movie showing the reactivity of an FCC particle close to the surface (Z = 0), exposed to 0.44 M solution of furfuryl alcohol in water. Reconstructed based on 1000 frames, exposure time of the EM-CCD is 0.75 ms per frame.

S1. Mechanism of the furfuryl alcohol self-condensation on Brønsted acid sites

S2. Absorption and fluorescence spectra of oligomeric species

S3. Experimental details

S4. Data analysis

S5. Thresholding and segmentation analysis

S6. SOFI intensity as a function of focal depth

S7. Confocal fluorescence microscopy study of the FCC particles

S8. Fluorescence intensity trajectories of individual zeolite domains

S1. Mechanism of the furfuryl alcohol self-condensation on Brønsted acid sites

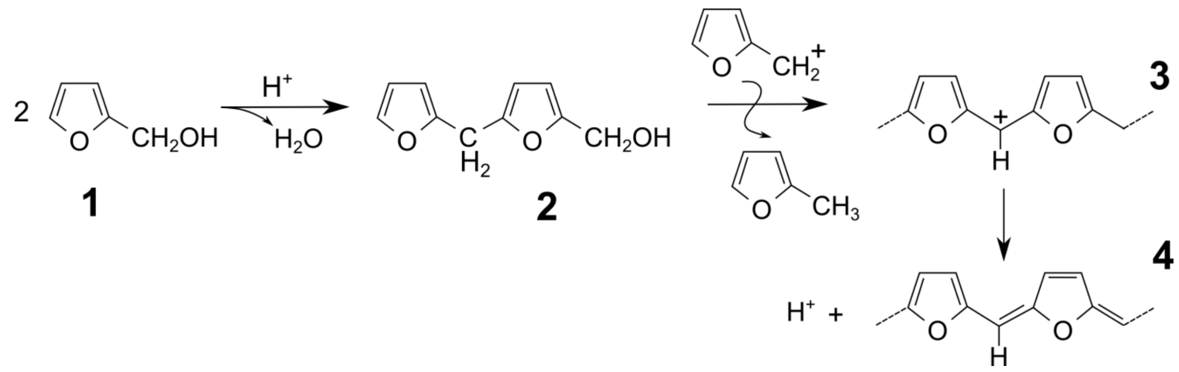

Figure S1. Formation of chromophores during the acid catalyzed condensation of furfuryl alcohol.[1] Initial protonation and dimerization of (**1**) leads to formation of non-colored bisfurylmethyl group (**2**). This molecule further undergoes hydride transfer to result in formation of resonance-stabilized carbenium ion (**3**) and its conjugated structure formed after proton loss (**4**). The carbenium ion (**3**) is reported to have an extinction coefficient of 55000 $cm^{-1}M^{-1}$ at 490 nm. Carbenium structure (**4**) has an estimated extinction coefficient of 110000 $cm^{-1}M^{-1}$ at 610 nm.[2]

S2. Absorption and fluorescence spectra of oligomeric species

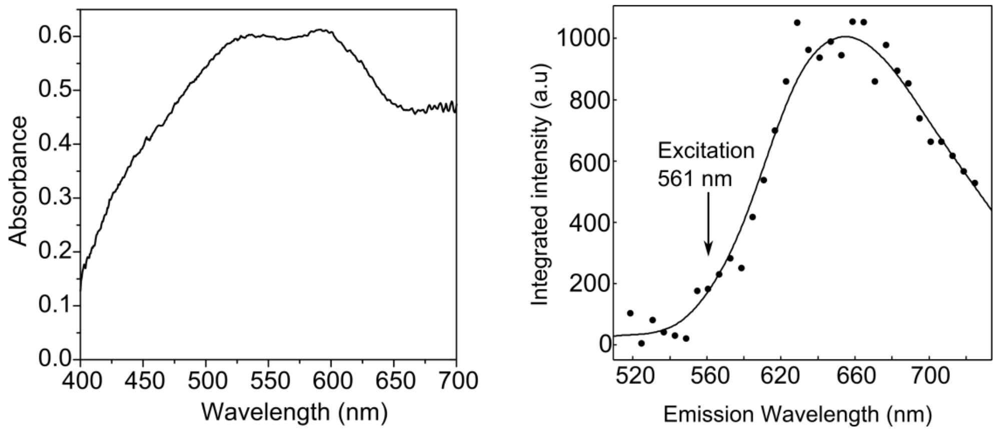

Figure S2. Absorption (left) and fluorescence spectra (right) of oligomeric species recorded at single FCC catalyst particles exposed to non-diluted furfuryl alcohol; Fluorescence spectrum is recorded using a confocal fluorescence microscope equipped with a spectral detection unit and a 561 nm laser excitation. Note that in the single molecule experiment we have used a 532 nm laser excitation.

S3. Experimental details

Sample preparation. Freshly prepared FCC particles based on zeolite ZSM-5 were provided by Albemarle Catalyst Company BV. The catalyst particles were thoroughly calcined in a static air oven at 823 K (heating ramp 0.5 K/min, 1 h dwell time at 353 K and 393 K) for 48 h prior to use in order to eliminate the residual fluorescence of impurities. Prior to the experiments the particles were spin-coated over cover slip and kept overnight in a static oven at 723 K (1 K/min, with 1 h dwell time at 393 K). The catalytic reaction is performed at room temperature in a reactor designed for a liquid-phase experiment using 0.44 M solution of furfuryl alcohol (Sigma Aldrich, 98 %) in water. The optimal concentration for imaging was determined in a series of concentration-dependent experiments. Prior to adding furfuryl alcohol the catalyst particles exhibited low residual fluorescence, which was additionally removed by 5 min of photobleaching with the intense laser light.

Experiment. Single molecule fluorescence experiments were performed using an inverted epi-fluorescence wide-field microscope (Olympus IX-71), with 100× oil immersion objective lens (1.4 NA). Wide-field illumination was achieved by circularly polarized 532 nm light from a diode-pumped solid-state laser (Excelsior 532 single mode 200 mW, Spectra-Physics), providing 25 mW power on the sample. Fluorescence emission is imaged by the EM-CCD (ImagEM Enhanced C9100-13) after passing through a dichroic mirror and a 545 nm long-pass filter removing the excitation light. The image was expanded by a 3.3× camera lens resulting in a field of view of 24.6×24.6 μm^2 and 48×48 nm^2 per pixel. Wide-field images were recorded with frame acquisition time of 75 ms.

S4. Data analysis

Recorded movies were analyzed using the Localizer software[3] (http://sushi.chem.kuleuven.be/localizer) developed for Igor Pro (Wavemetrics) and Matlab (MathWorks). Sub-diffraction localization of fluorescent events was done by independent segmentation of each frame into emissive spots and background using the approach of Serge et al.[4] that was implemented using the FFTW library (http://www.fftw.org/). Subsequent fitting of single emitters with a two-dimensional Gaussian was done using the Levenberg-Marquardt least-squares algorithm as implemented in the GNU Scientific Library (http://www.gnu.org/software/gsl/). SOFI images were analyzed with Localizer software using a second-order cross-correlation based on previously published algorithms.[5,6]

S5. Thresholding and segmentation analysis

Images depicting the intensity of all pixels after SOFI analysis were loaded into Matlab where they were cropped to solely display the area of interest. A circular crop was then applied to remove the inner regions of the particle with lower SOFI intensities. This simultaneously minimizes differences in depth (due to the spherical nature of the catalysts particle) and related intensity differences due to absorption effects. The image was then converted into a binary, black-white (BW) image, using a threshold and a gray scale image. Thresholds were chosen based upon the amount of detected (white) pixels. This was done by analyzing thresholds between 0 and 1 (Figure S3a-c) and selecting the optimal threshold, which was also verified by visual inspection of bright domains. Low threshold values (< 0.2) typically lead to the clustering and oversizing of zeolite domains while high values (> 0.3) underestimate the total fluorescent area (Figure S3d). The isolated pixelated areas of 1 pixel in size were then removed and holes filled in the BW image. The gray scale image and the resulting BW image were then loaded into the 'imageprops' Matlab (MathWorks) routine, which analyzed the area of all white domains on a black background. Finally, histograms of size distribution in Figure 3 are reconstructed based on chosen thresholds. The domains smaller than 0.01 μm^2 (2×2 pixel areas) were not included in the histograms.

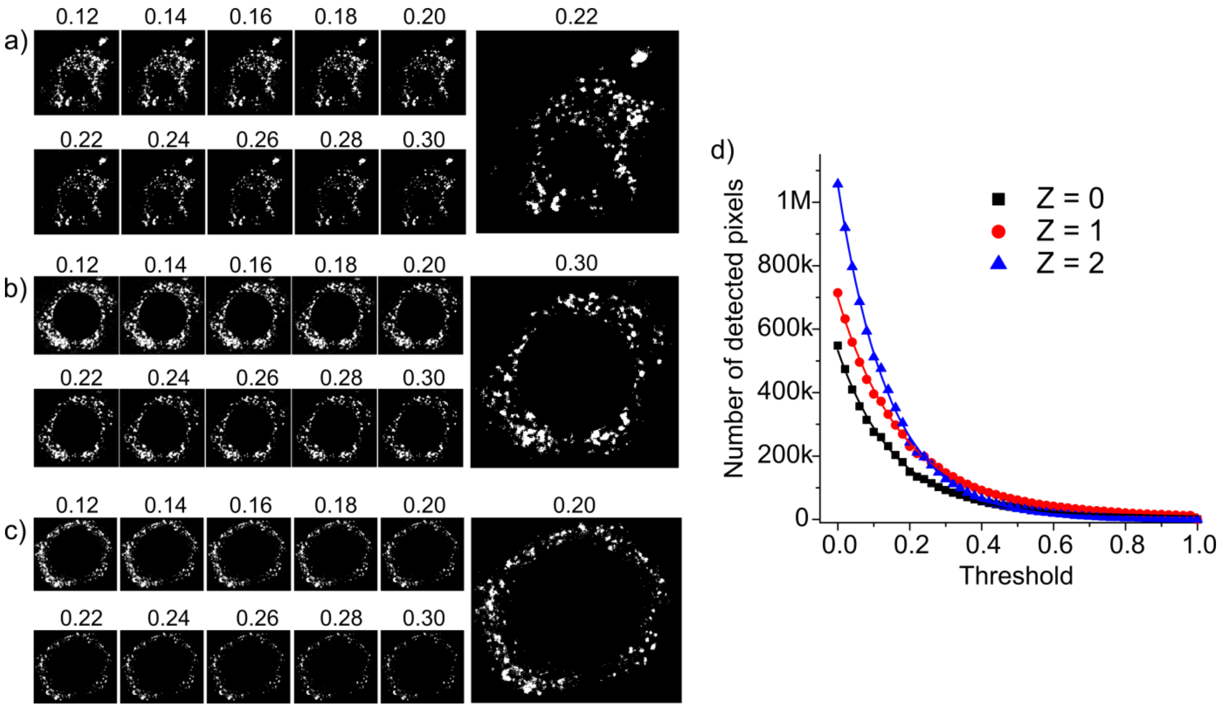

Figure S3. Thresholding procedure applied to SOFI images from Figure 3. a) Z = 0, surface, b) Z = 1 µm, c) Z = 2 µm. Thresholding image series from S3a and S3c are reconstructed based on 10000 frames movie, while thresholding image series S3b was reconstructed based on 5000 frames movie. An optimum thresholding values of a) 0.22, b) 0.30, and c) 0.2 were found to represent well bright zeolite domains. The values differ due to differences in SOFI intensities that have an effect on the segmentation procedure. Relative error for calculated size of zeolite domains is ~ 10%, estimated based on the total number of detected pixels. d) The number of detected pixels above a certain threshold.

S6. SOFI intensity as a function of focal depth

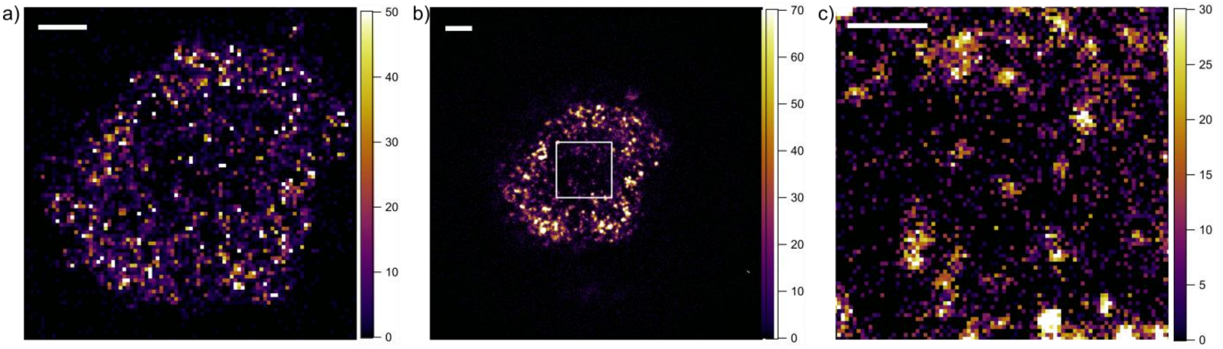

Figure S4. a) Binned NASCA image (144×144 nm^2 per pixel), and b) corresponding SOFI image, illustrating higher fluorescence activity in the outer region of an FCC particle; c) Magnified region of interest from b, illustrating lower SOFI intensities for inner zeolite domains. Scale bars are 2 μm.

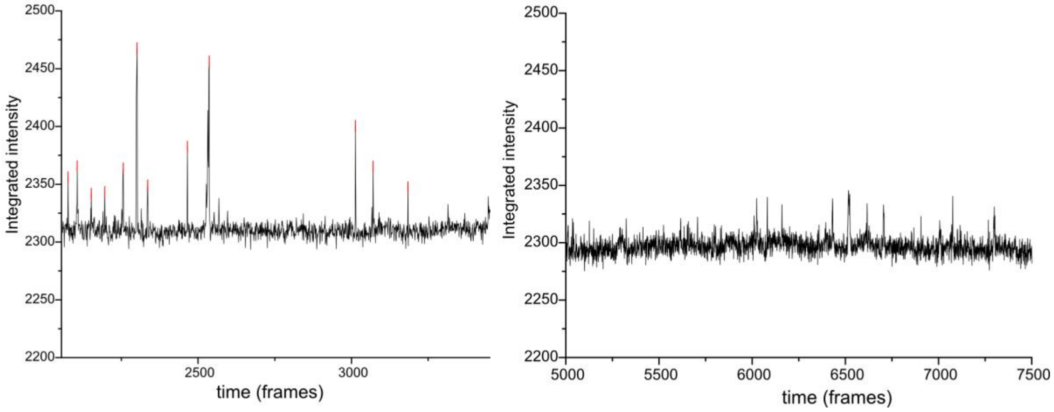

Figure S5. Typical fluorescent trajectories recorded at Z = 0 (left) and at Z = 2 μm (right). Note the attenuation of fluorescent events at higher focal depth that results in lower values of SOFI intensity.

S7. Confocal fluorescence microscopy study of the FCC particles

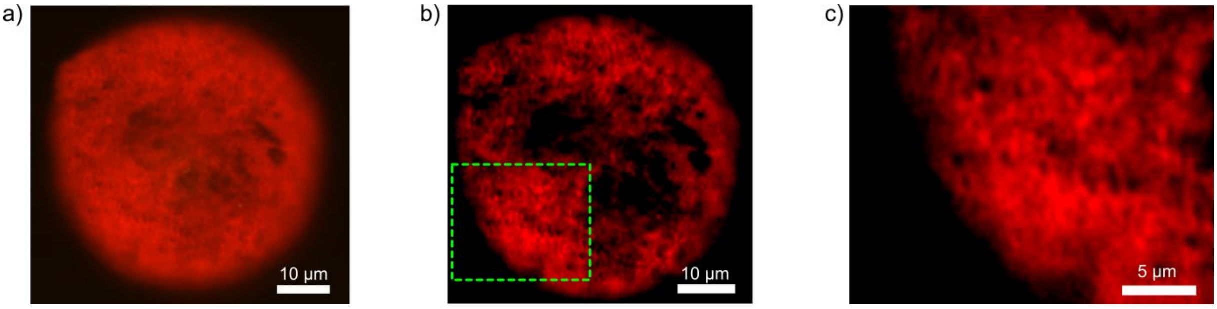

Figure S6. Confocal fluorescence images of an FCC particle recorded after exposure to non-diluted furfuryl alcohol, focal depth ~ 3 μm. a) Raw image of fluorescence intensities, b) Rescaled image of fluorescence intensities, c) Magnified region from b) showing zeolite particulates smaller than 1 $μm^2$.

S8. Fluorescence intensity trajectories of individual zeolite domains

Using a SOFI image from Figure 3a of the main manuscript, 65 fluorescence intensity trajectories of individual domains within ~ 6×6 μm^2 region of interest were constructed by integrating the fluorescence intensity of 3 × 3 pixelated regions (~ 144×144 nm^2). The number of catalytic turnovers was then determined from fluorescence intensity trajectories, as each spike in the trajectory represents one fluorescent (catalytic) event. Single turnovers were detected using a Peak Analyser routine, implemented into Origin 8.1. Local peaks are also checked manually, to verify correctness of the algorithm. Turnover rate was further calculated by normalizing the number of detected turnovers per unit of time and analyzed area.

Similar integration of the averaged SOFI intensity was done for SOFI images using 3×3 pixel ROIs. This way a plot in Figure 4e was constructed.

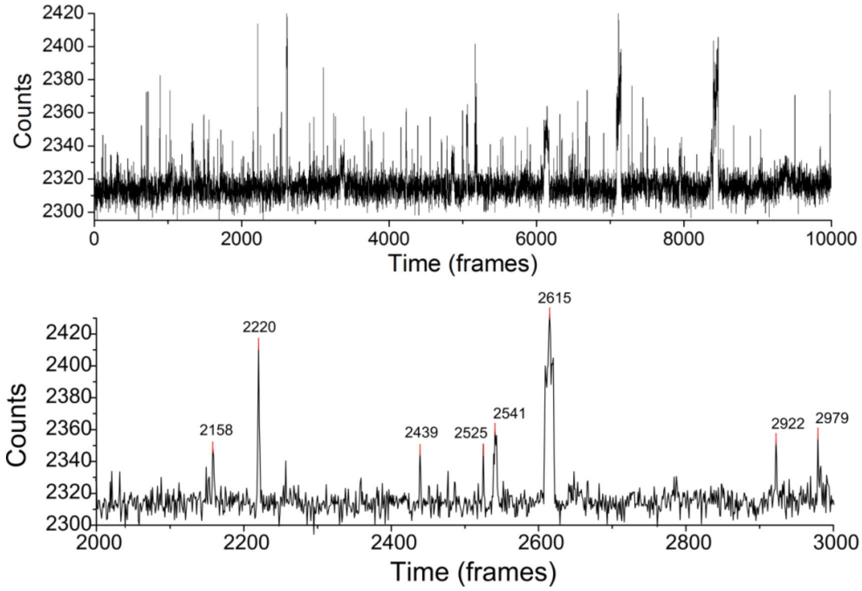

Figure S7. Top: A fluorescence intensity trajectory of a single zeolite domain reconstructed by integrating fluorescence signal over 3 × 3 pixelated region of interest. Bottom: a zoom in from the top trajectory, indicating assigned single catalytic turnovers. 1 frame = 75 ms.

Supporing references

[1] M. B. J. Roeffaers, G. De Cremer, J. Libeert, R. Ameloot, P. Dedecker, A.-J. Bons, M. Bückins, J. A. Martens, B. F. Sels, D. E. De Vos, et al., *Angew. Chem.* **2009**, *121*, 9285–9289.

[2] M. Choura, N. M. Belgacem, A. Gandini, *Macromolecules* **1996**, *29*, 3839–3850.

[3] P. Dedecker, S. Duwé, R. K. Neely, J. Zhang, *J. Biomed. Opt.* **2012**, *17*, 126008–126008.

[4] A. Sergé, N. Bertaux, H. Rigneault, D. Marguet, *Nat. Methods* **2008**, *5*, 687–694.

[5] T. Dertinger, R. Colyer, G. Iyer, S. Weiss, J. Enderlein, *Proc. Natl. Acad. Sci.* **2009**, *106*, 22287–22292.

[6] T. Dertinger, R. Colyer, R. Vogel, J. Enderlein, S. Weiss, *Opt. Express* **2010**, *18*, 18875.
